# Managing Fear and Anxiety in Patients Undergoing Dental Hygiene Visits with Guided Biofilm Therapy: Analysis of Psychological and Physiological Differences Between Women and Men—A Conceptual and Multivariate Regression Model

**DOI:** 10.3390/jpm15040147

**Published:** 2025-04-08

**Authors:** Marta Leśna, Krystyna Górna, Jakub Kwiatek

**Affiliations:** 1 Kwiatek Dental Clinic, Kordeckiego 22, 60-144 Poznań, Poland; jakubkwiatek@klinikakwiatek.pl; 2 Department of Psychiatric Nursing, Poznan University of Medical Sciences, Rokietnicka 2A, 60-806 Poznań, Poland; gorna@ump.edu.pl

**Keywords:** dental anxiety, dental fear, gender differences, Guided Biofilm Therapy (GBT), AI Diagnocat, anxiety management, conceptual model, anxiety reduction strategies, patient-centered dental care, Modified Dental Anxiety Scale (MDAS), State-Trait Anxiety Inventory (STAI)

## Abstract

**Background:** Dental anxiety is a significant barrier to dental care, leading to avoidance behaviors and compromised oral health. This study aimed to analyze fear and anxiety during dental hygiene visits with Guided Biofilm Therapy (GBT), focusing on gender differences in psychological and physiological responses to develop a more personalized approach to dental care. **Methods:** A total of 247 patients participated in this study. Psychological assessments included the Modified Dental Anxiety Scale (MDAS) and the State-Trait Anxiety Inventory (STAI X2), while physiological responses were measured through heart rate monitoring before and after procedures. Multivariate regression analysis was conducted to identify predictors of anxiety levels. **Results:** Multivariate regression analysis identified gender, sensory sensitivity (e.g., absence of tools in the field of view), past traumatic dental experiences, and individual preferences for anxiety reduction as significant predictors of anxiety levels. Gender differences were also observed in anxiety management strategies, with women more frequently preferring the elimination of sensory triggers and direct communication with dental professionals. **Conclusions:** The findings highlight the importance of personalized anxiety management protocols in dentistry. Tailored communication strategies, optimized clinical environments, and individualized pre- and post-procedure care plans can enhance patient experience and treatment acceptance. Implementing such patient-centered, data-driven approaches aligns with the broader principles of precision medicine in dental care.

## 1. Introduction

### 1.1. Background and Significance of the Problem

Dental fear and anxiety are common issues, with studies reporting varying percentages of individuals affected [1]. In Western countries, dental anxiety has been estimated at over 10% in a study by McGrath and Bedi [2]. Other studies indicate that the prevalence ranges from 4% to 20% in the general population of industrialized countries [3]. In contrast, high dental anxiety is reported in most available studies to affect fewer than 10% of the population [4]. Despite advancements in technology and treatment methods, many patients continue to experience significant levels of fear and anxiety before dental visits, often leading to the avoidance of regular check-ups. This avoidance can result in the deterioration of oral health, which in turn negatively affects overall health, including cardiovascular and metabolic conditions [5]. It is a phenomenon with serious consequences that requires a thorough approach to help patients break the cycle of avoiding visits, as indicated by most researchers [6].

In response to these challenges, modern treatment and preventive protocols such as Guided Biofilm Therapy (GBT) have been developed [7,8].

GBT is a minimally invasive hygiene method that focuses on effective biofilm removal while reducing patient discomfort through advanced technologies. These technologies include ultrasonic scaling with “no-pain” technology and biofilm detection systems, which help minimize procedure duration and improve the overall patient experience [7,8]. The process is fully monitored, and patients have the opportunity to track their home hygiene progress from visit to visit, which may contribute to reducing their levels of fear and anxiety. Including GBT in this discussion provides a contextual framework for modern biofilm management protocols and illustrates the shift towards patient-centered, minimally invasive dental care.

Research suggests that implementing GBT as the standard in preventive and hygiene procedures can significantly reduce fear and anxiety related to dental treatments [8]. This, in turn, can help patients break the “vicious cycle” of dental anxiety, describing the continuously increasing health and psychological issues associated with avoiding dental care.

Studying patients’ feelings of fear and anxiety during the use of GBT is particularly important, as this method is gradually replacing traditional, more invasive oral hygiene techniques. With the growing popularity of this technique, which is replacing manual scaling and other older procedures, it is crucial to understand how patients react to treatments using GBT, especially in terms of dental fear and anxiety. Considering the individual differences in patients’ perception of pain, anxiety, and physiological responses, standard approaches that do not account for psychological factors and gender disparities may not be optimal for all individuals. Our study provides data that support the development of precise anxiety reduction strategies, tailored to gender, medical history, and patient preferences. A more personalized approach in dental care could improve treatment adherence and patient outcomes by addressing specific anxiety triggers and enhancing comfort during procedures. This knowledge can help further refine methods to reduce anxiety and improve patient comfort, which may encourage more regular participation in hygiene treatments and lead to an overall better quality of life.

The issues of dental fear and anxiety are currently the subject of many studies and literature reports. This is a complex phenomenon, influenced by numerous factors that often correlate with one another. Economou emphasizes the importance of self-awareness in shaping dental anxiety [1]. On the other hand, Yokota et al., in their study of the Australian homeless population, highlight the key role of feelings of shame and the belief in having poor oral health [9].

Studies by Ogawa et al. point to the relationship between sensory sensitivity and pain catastrophizing [10]. Vassend et al., in their study on twins, analyze the genetic and environmental influences on neuroticism and pain perception as factors contributing to susceptibility to dental anxiety [6].

However, it is important to consider the role of non-modifiable factors such as the patient’s gender, as highlighted by Goh et al. in their literature review analyzing anxiety related to dental visits [11]. Gender differences influence dental fear and anxiety, necessitating the development of tailored management protocols for dental visits.

Research indicates that women often report higher levels of anxiety and fear before dental visits compared to men, which may be partly attributed to hormonal differences and social factors related to health perception [12,13].

Conversely, men may experience and express dental anxiety differently, potentially concealing discomfort, which can complicate accurate assessments of their needs [13]. Understanding these differences is crucial when applying modern technologies like GBT, as it provides an opportunity to develop individualized strategies that address specific psychological and physiological responses to dental procedures. Gender-based analysis of these responses can enhance protocol effectiveness, improving the patient experience for both women and men [14].

### 1.2. Research Objective

The objective of this study is to assess anxiety levels in men and women during hygiene visits involving Guided Biofilm Therapy (GBT). Specifically, the study focuses on analyzing the influence of gender on various anxiety-related parameters. When a significant association between gender and specific anxiety variables is observed, the study is expanded to include a multivariate regression model to determine whether gender is a significant predictor of anxiety levels. The findings may provide valuable insights for personalizing hygiene protocols and improving care for patients of both genders. We hypothesized that gender would significantly influence anxiety-related outcomes, with women expected to report higher levels of anxiety and pain.

## 2. Materials and Methods

The study was cross-sectional and prospective in nature, and its protocol was approved by the Bioethics Committee of the Poznan University of Medical Sciences. Inclusion and exclusion criteria were carefully designed to ensure objectivity and patient diversity. Patients included in the study were aged 18 years and above, able to provide informed consent, and scheduled for oral hygiene procedures using the Guided Biofilm Therapy (GBT) method between September 2023 and January 2024 at Kwiatek Dental Clinic in Poznań (Poland). Additional inclusion criteria required recent radiographs to be available and the ability of participants to provide complete responses to the study questionnaires. Radiographs were used to assess key clinical conditions in the oral cavity (e.g., caries, root canal treatments, implants, periodontal disease), and this assessment was additionally confirmed using the Diagnocat AI system. Exclusion criteria included individuals under 18 years of age, those unable to provide consent, legally incapacitated individuals, and patients with incomplete data or missing radiographic records. Participants with self-reported mental health problems (e.g., anxiety, depressive symptoms) were not excluded from the study, as they were capable of providing informed consent and completing the questionnaires independently.

The procedures were performed by three dental hygienists with comparable experience, following uniform standards of care. This study focuses on the differences in the experience of fear and anxiety between genders. The analysis included a comparison of fear and anxiety levels (measured using the MDAS and STAI X1/X2 before the hygiene procedure; author-developed questions and the Gatchel scale were used both before and after the procedure) between women and men, taking into account sociodemographic and clinical factors, including those obtained using the AI Diagnocat system. Clinical and contextual factors were also assessed based on participant responses. These included self-reported pain intensity during the hygiene visit (measured using a Visual Analogue Scale), medical history, frequency of dental visits, and selected situational elements (e.g., the behavior of the hygienist, communication style, or environmental comfort). These variables were used to explore their relationship with anxiety and pain perception. A more detailed description is available in a previous publication [14].

### 2.1. Study Sample

The study included 247 patients who consecutively underwent hygiene visits. The inclusion and exclusion criteria are described in this article, while further participant characteristics were detailed in a previous publication [14].

However, for clarity, key demographic data are also presented here. The study included 247 adult participants (147 women, 100 men), aged 19–76 years (mean age: 43.52 ± 12.95 years).

### 2.2. Research Procedure

The research procedure included an examination of the patient before, during, and after the hygiene procedure. It was described in detail in the article by Leśna et al. [12]. Measurement tools and techniques were employed, including questionnaires assessing anxiety levels and devices monitoring physiological responses. The measures of fear and anxiety used in this study were standardized questionnaires: the MDAS (Modified Dental Anxiety Scale) assessing dental anxiety, the STAI (State-Trait Anxiety Inventory) X1, which analyzes state anxiety, and X2, which assesses trait anxiety, as well as the Gatchel scale for assessing dental fear. Additionally, a custom question regarding the patient’s fear related to the hygiene procedure was used. Among the clinical and physiological factors examined were heart rate, oral health parameters, and health issues. Sociodemographic factors, as well as those that could reduce the experience of negative emotions (fear, anxiety) from the patient’s perspective, were also considered. The research tools were described in detail in a previous article [14].

### 2.3. Statistical Analysis

Statistical analyses were performed using Statistica software, version 13. Due to the sample size and potential deviations from normality, non-parametric tests (Mann–Whitney U test, χ^2^ test) were applied for comparisons between groups. Descriptive statistics included means, standard deviations (SD), medians, interquartile range, and 95% confidence intervals (CI). The following analyses were conducted: Chi-square test (Chi^2^), Spearman’s rank correlation, Student’s *t*-test, Friedman test, ANOVA (Analysis of Variance), Mann-Whitney U test, and Wilcoxon test. Additionally, multivariate regression models were constructed to identify predictors of dental anxiety (MDAS) and trait anxiety (STAI X2). The values of beta coefficients (β), determination coefficient (R^2^), and F-statistics were used to assess the quality and strength of the models. Statistical significance was determined based on *p*-values, with *p* < 0.05 considered statistically significant.

## 3. Results

### 3.1. Characteristics of the Study Group

**Figure 1:**
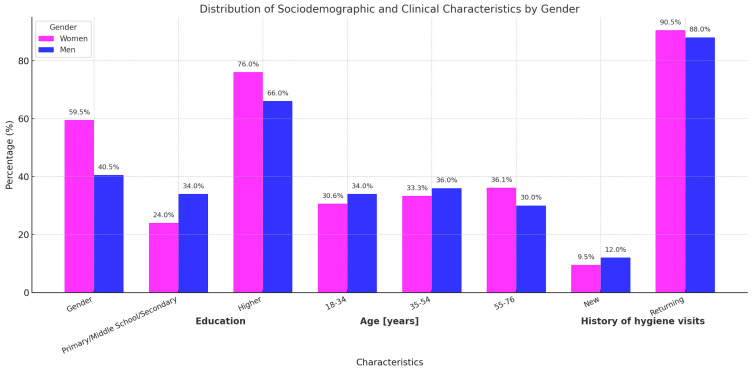
Characteristics of the study sample.

The study group consisted of 247 participants. Detailed sociodemographic characteristics have been presented in a previous publication [14]. This analysis focuses specifically on gender-based differences in anxiety-related parameters, as shown in Table 1 and in the chart (Figure 1).

The variable values, including a breakdown by gender (women and men), are presented in Table 2.

### 3.2. Psychological Reactions

#### 3.2.1. Dental Anxiety (MDAS)

**Figure 2:**
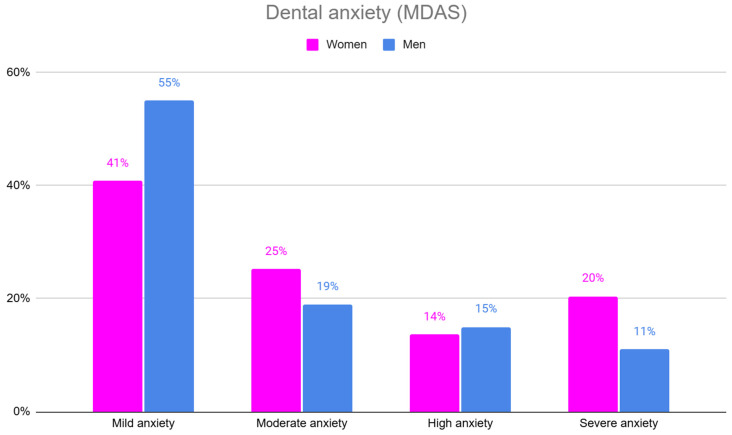
The level of dental anxiety (MDAS) in women and men.

Significant differences in dental anxiety (MDAS) were observed between women and men, with women reporting higher levels of moderate and severe anxiety compared to men (Table 3, Figure 2).

#### 3.2.2. Trait Anxiety (STAI X2)

**Figure 3:**
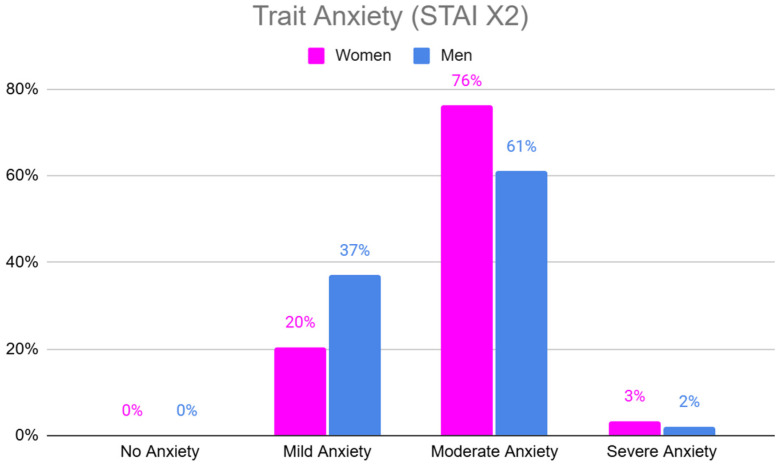
Trait Anxiety levels (STAI X2) in women and men.

Trait anxiety (STAI X2) was significantly higher among women compared to men, with a greater proportion of women reporting moderate levels of anxiety compared to men (Table 4, Figure 3).

**Figure 4:**
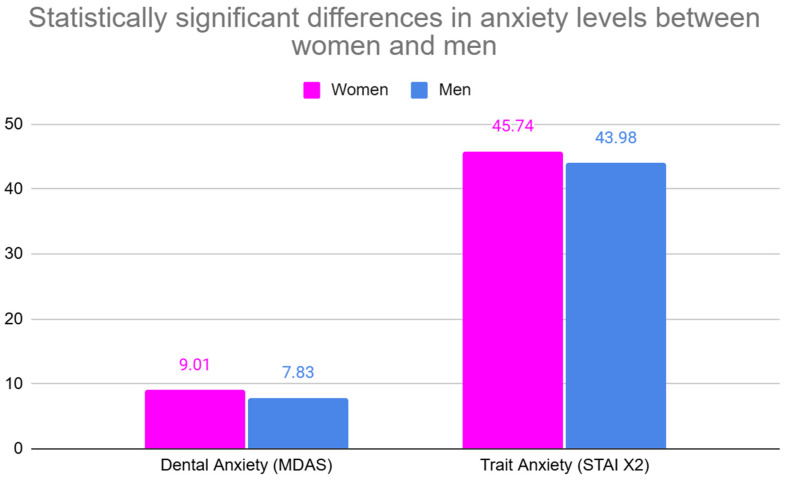
Levels of Dental Anxiety (MDAS) and Trait Anxiety (STAI X2) in women and men. All comparisons are statistically significant.

Figure 4 illustrates the statistically significant differences in trait anxiety (STAI X2) between women and men, alongside dental anxiety (MDAS), highlighting consistently higher anxiety levels reported by women across both measures.

#### 3.2.3. Variables Without Statistically Significant Differences

No statistically significant differences were observed for the following variables: state anxiety (STAI X1), dental fear (Gatchel’s Scale), and fear related to the hygiene procedure. These results are summarized in Table 2.

### 3.3. Physiological Responses–Heart Rate

**Figure 5:**
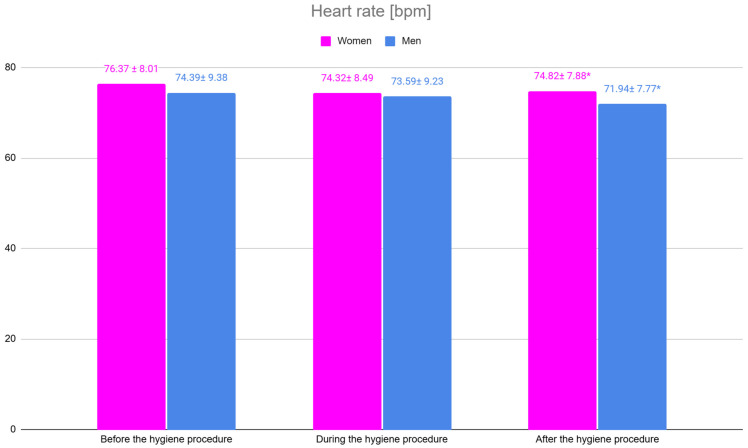
Heart rate levels before, during and after hygiene procedures in women and men. * *p* < 0.05.

Heart rate measurements revealed statistically significant differences between women and men after the hygiene procedure, with women demonstrating a higher mean heart rate (74.82 bpm) compared to men (71.94 bpm). No significant differences were noted before or during the procedure. These results are presented in Table 2 and illustrated in Figure 5, which shows heart rate measurements at all three time points: before, during, and after the hygiene procedure.

### 3.4. Factors Related to Hygiene Visits That, in the Patient’s Opinion, Could Reduce the Experience of Negative Emotions (Fear, Anxiety)

In the conducted study, patients evaluated factors that, in their opinion, could reduce negative emotions associated with hygiene visits. Statistically significant differences between women and men were observed for several factors, including the absence of tools in the field of view, the use of sedatives and painkillers, and the possibility of contacting the clinic/dentist/hygienist. The test values and significance levels (*p*-values) are presented in Table 5.

### 3.5. Health Differences Between Women and Men

In the conducted study, significantly higher clinical factors were observed in women compared to men. The test values and significance levels (*p*-values) are presented in Table 6, showing significant differences in areas such as physical health problems, mental health problems, and the use of various medications, including those for depression, insomnia, and other mental disorders.

Women were found to take medications significantly more often than men, particularly thyroid medications, psychotropic drugs for mental health issues, and “other” medications. The reported mental health problems included depression, anxiety disorders, depressive episodes, anxious-depressive disorders, sleep disturbances, emotional low moods (“feeling down”), and symptoms consistent with emotional post-traumatic stress disorder (ePTSD).

These differences are detailed in Table 6.

Table 7 presents the relationship between medication use and anxiety levels (MDAS and STAI X2). The analysis indicates a potential connection between certain medications and higher anxiety levels, with specific differences observed for “other” medications in relation to trait anxiety (STAI X2). However, no statistically significant association was observed for thyroid medications or medications for depression and insomnia in the context of dental anxiety (MDAS).

### 3.6. Regression Analysis for Trait Anxiety (STAI X2)

A multivariate regression model was developed to identify variables influencing the level of trait anxiety (STAI X2). Independent variables included in the model were those significantly associated with the dependent variable and meeting inclusion criteria, such as fear related to hygiene procedures assessed before the procedure (r = 0.13, *p* = 0.0397), gender (t = −3.37, *p* = 0.0009), taking medications (t = −4.37, *p* < 0.0001), and factors reducing negative emotions in the patient’s opinion, such as the absence of tools in the field of view (r = 0.17, *p* = 0.0081) and the sense of comfort in the dental chair (r = 0.13, *p* = 0.0400).

Beta coefficients (β) were used to compare the relative contributions of each independent variable to the prediction of trait anxiety (STAI X2). The strongest predictors of trait anxiety were taking medications, gender, and the absence of tools in the field of view, with only the first two being statistically significant. The final regression model (Table 8) (F(3,243) = 10.19, *p* ≤ 0.0001) moderately explained the variance of trait anxiety (R^2^ = 11%). This level of variance is considered acceptable in social and behavioral research, particularly in multivariate analyses, as suggested by Falk and Miller [15].

### 3.7. Regression Analysis for Dental Anxiety (MDAS)

To identify variables influencing the level of dental anxiety (MDAS), a multivariate regression model was constructed. The model included only significantly associated independent variables that met the inclusion criteria: gender (t = −2.59, *p* = 0.0102), frequency of dental visits (t = −3.33, *p* = 0.0010), oral hygiene self-assessment (t = −4.60, *p* < 0.0001), mental health issues (t = −2.26, *p* = 0.0249), taking medication (t = −2.42, *p* = 0.0164), traumatic dental experiences in the past (t = −4.83, *p* < 0.0001), and factors that, in the patient’s opinion, could reduce negative emotions, such as absence of tools in the field of view (t = 5.18, *p* < 0.0001), absence of sounds from working tools (t = 3.45, *p* = 0.0007), absence of characteristic dental office smells (t = 3.94, *p* = 0.0001), possibility of using sedatives and painkillers (t = 2.99, *p* = 0.0030), pain experienced during hygiene procedures (t = 5.35, *p* < 0.0001), and number of carious lesions (t = 2.73, *p* = 0.0069). The Beta coefficients (β) reflect the relative contributions of each independent variable to the prediction of the dependent variable (MDAS). The most significant predictors of dental anxiety (MDAS) were pain experienced during hygiene procedures, absence of traumatic dental experiences in the past, absence of sounds from working tools, frequency of dental visits, number of carious lesions, good oral hygiene self-assessment, and absence of mental health issues. All variables, except for gender and not taking medication, were statistically significant. The final regression model (Table 9) (F(8,238) = 13.37, *p* ≤ 0.0001) explained 31% of the variance in the dependent variable MDAS (R^2^ = 31%). According to the criteria of Falk and Miller [13], this result demonstrates good model validity and its applicability in explaining the phenomenon of dental anxiety in the context of social and behavioral research.

## 4. Discussion

### 4.1. Differences in Psychological Reactions Between Women and Men Undergoing Hygiene Visits

The results of this study confirm the existence of differences in psychological and physiological responses between women and men undergoing hygiene procedures using Guided Biofilm Therapy (GBT) technology. Women exhibited higher levels of dental anxiety (MDAS) and trait anxiety (STAI X2) compared to men. These findings are consistent with studies reported in the literature [12,16,17]. Notably, Yakar et al. conducted a study utilizing both of these parameters and obtained results consistent with the present analysis [18].

Trait anxiety (STAI X2) refers to a stable, long-term tendency to experience anxiety in various situations. It is a consistent inclination to react with anxiety, regardless of circumstances, which can be interpreted as a personality predisposition that affects how a person perceives and responds to the surrounding world. Women, in many studies, exhibit higher levels of this trait, which may be due to biological, hormonal, and social factors [19].

State anxiety (situational anxiety) (STAI X1), on the other hand, refers to a short-term reaction to a triggering situation (in this case, the hygiene procedure). The small and statistically insignificant difference in situational anxiety between women and men (before and after the procedure) may indicate that, despite higher overall levels of anxiety, women may react similarly to men in a specific dental situation. A similar result was obtained in a study conducted by Razavian et al. [20].

The literature offers several explanations for these findings. Despite having higher trait anxiety, women may be better mentally prepared for dental situations and adapt to them more effectively. This could be due to previous dental experiences, regular visits, or more effective coping mechanisms for anxiety in this specific context [21,22,23]. Frequent utilization of dental services may help women anticipate the procedure, which, in turn, could lower their situational anxiety.

Social conditioning might also play a role in the experience of anxiety. Women may be more aware of or accustomed to experiencing anxiety as a personality trait, which leads to higher anxiety tendencies. However, during a specific situation like a hygiene procedure, they might focus more on the task at hand, enabling them to manage situational anxiety at a similar level to men [24].

Hormonal differences, such as those involving estrogen and progesterone, may contribute to the higher trait anxiety scores seen in women [25]. At the same time, stress hormones like cortisol might act similarly in both sexes during short-term stressful situations, such as a dental procedure, leading to comparable results on the STAI X1 scale.

Women may also possess more effective emotional regulation mechanisms and greater behavioral flexibility during specific stressors, such as dental visits, which could help them cope with situational anxiety more effectively [26]. In summary, higher trait anxiety (STAI X2) in women reflects their general tendency to experience anxiety, while the lack of differences in situational anxiety (STAI X1) may be related to learned coping strategies for specific stressful situations.

For clinical practice, these findings are particularly important for both dentists and dental hygienists. They show that even though women may report higher general anxiety, in specific dental situations like hygiene procedures, they can cope equally well. This emphasizes the need for personalized patient communication and creating a calm, reassuring environment. Regular exposure, clear explanations, and an empathetic approach by the dental team may help reduce anxiety, especially in female patients.

#### The Impact of the COVID-19 Pandemic on Dental Anxiety

External public health crises, such as the COVID-19 pandemic, have been shown to influence dental anxiety levels. A study conducted in Poland during the pandemic revealed that despite implemented safety protocols, many patients continued to experience elevated anxiety related to dental visits [27]. Over 70% of respondents reported moderate to high levels of fear, with key concerns including fear of infection, limited access to care, and increased treatment costs.

Interestingly, while this study did not find statistically significant gender differences in COVID-related dental anxiety, the reported fear levels were slightly higher among women. This aligns with broader literature suggesting that women are generally more reactive to pandemic-related stressors [28,29]. The absence of statistical significance may reflect sample size or context-specific factors, but the trend is consistent with gender-based patterns of anxiety observed in dental settings before and after the pandemic.

These findings indicate that the pandemic may have exacerbated existing patterns of dental fear, with implications for long-term patient behavior and gender-sensitive management strategies.

### 4.2. Differences in Physiological Reactions Between Women and Men Undergoing Hygiene Visits

Heart rate measurements indicated significant differences between women and men, both before and after the procedure. Women exhibited higher heart rate values, suggesting a stronger emotional response to anxiety. While these differences were statistically significant, the 2 bpm difference, with a standard deviation between 7 and 10, may be considered marginal in biological terms. Nevertheless, even small differences in heart rate could reflect a heightened stress response in women, which is consistent with their higher trait anxiety levels.

Resting heart rates ranging from 60 to 100 bpm are considered normal for adults, and studies indicate that women generally have higher average heart rates than men. This observation aligns with research by Bangasser et al., who, using an animal model, found that the female sex has a greater susceptibility to dysregulation after a stressful event. They attribute this to physiological processes involving weaker glucocorticoid receptor function and slower negative feedback mechanisms [30].

Men may return to a state of equilibrium more quickly after stressful experiences, while women tend to calm their nervous system more slowly, which could explain the prolonged elevated heart rate observed after the procedure.

Other potential factors include environmental and contextual influences. Women may be more sensitive to their surroundings and experience greater discomfort due to stimuli during and after the procedure. Concerns about procedural outcomes may also influence physiological responses, such as heart rate.

In daily practice, recognizing that some patients—especially women—may experience physiological signs of stress despite appearing calm can help both dentists and dental hygienists provide more empathetic, tailored care during hygiene visits.

### 4.3. Differences in the Assessment of Factors That Could Reduce the Experience of Negative Emotions in Women and Men

Women more strongly than men preferred the elimination of sensory stimuli during the hygiene procedure. They rated the absence of tools in their field of vision, the lack of sound from working instruments, and the absence of characteristic dental office smells as more important. These findings align with previous studies indicating that women more often experience discomfort related to noise, smells, and the sight of dental tools [24,31,32].

Women also rated the use of sedatives and pain relievers, as well as the option of taking breaks during the procedure, higher than men. This is consistent with the findings of Liddell and Locker, who demonstrated that women more frequently report higher anxiety related to the lack of control during dental procedures [33].

Additionally, women indicated a stronger preference for post-procedure contact with medical staff. Studies confirm that women value communication and access to health-related information more than men, especially in the context of anxiety related to medical procedures. Marca-Frances et al., for example, found that women initiated significantly more messages and inquiries after outpatient surgical visits [34].

These findings highlight actionable steps that dental professionals—especially hygienists—can take to reduce anxiety: minimizing sensory stimuli, allowing for pauses, and offering post-procedural communication when needed, particularly for women.

### 4.4. Health-Related Factors That May Modify the Experience of Negative Emotions in Women and Men

The data show that women more frequently report somatic health issues (32.65% vs. 20%) and mental health issues (10.88% vs. 2%) compared to men. This aligns with findings in the literature [35,36]. Women also more frequently take medications, including thyroid medications (17.01% vs. 2%) and psychotropic drugs (9.52% vs. 2%), which may influence their experience of negative emotions. These health-related factors appear to more strongly modify anxiety levels in women.

These findings emphasize the value of brief health-related screening or conversation before treatment, especially with female patients. Dental hygienists and dentists can better support patient comfort by being attentive to underlying conditions that may intensify anxiety responses.

### 4.5. The Value of Multivariate Models for Dental Practice

The multivariate analyses for the STAI X2 and MDAS models highlight the importance of various factors in modifying dental fear and anxiety. Gender was an explanatory variable in both models, enhancing their predictive accuracy and illustrating differences in emotional responses during dental visits. For the STAI X2 model, factors such as medication use and the absence of tools in the field of view contributed to an R^2^ of 11%. In the MDAS model, variables including traumatic dental experiences, the absence of sounds from working tools, and the frequency of dental visits resulted in an R^2^ of 31%.

These findings underscore the utility of multivariate models in identifying predictors of dental anxiety and highlight their role in personalized dental care. By integrating precision medicine principles, these models allow practitioners to develop tailored interventions based on individual patient profiles, including gender, past traumatic experiences, and sensory sensitivities.

Personalized strategies could include gender-sensitive approaches—for example, limiting sensory stimuli such as tool visibility and sound reduction for female patients, while prioritizing structured communication and predictability for male patients. Such data-driven, patient-centered interventions may enhance treatment adherence and reduce dental anxiety more effectively than generalized protocols. The incorporation of individualized risk assessment tools into routine dental practice may further refine patient management strategies, allowing for early identification of high-risk individuals and preemptive intervention, ultimately aligning with the broader shift toward precision dentistry.

Dental hygienists, in particular, can play a key role in implementing these personalized strategies during routine visits, helping reduce anxiety through attentive communication and sensitivity to patient needs.

### 4.6. Conceptual Model and Multivariate Regression Model for Managing Fear and Anxiety in Patients Undergoing Dental Hygiene Visits, Considering Gender Differences

**Figure 6:**
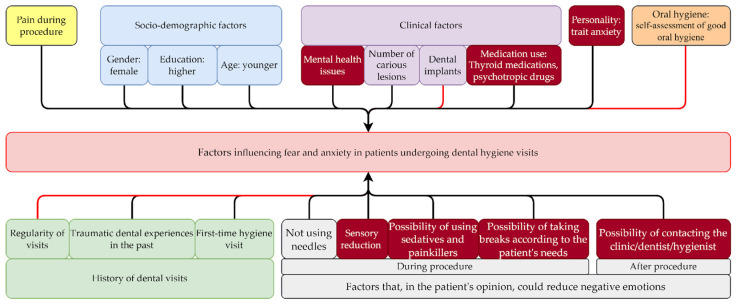
Conceptual model of the influence of socio-demographic, clinical, and patient experience factors on the level of fear and anxiety during a hygiene visit and strategies for their reduction. Dark red tiles represent factors that have a significantly stronger impact on women (red arrows: negative correlation, black arrows: positive correlation).

The obtained data were applied to the previously developed conceptual model illustrating the study on fear and anxiety in patients undergoing dental hygiene visits (Figure 6). This model outlines the influence of socio-demographic, clinical, and patient experience factors on fear and anxiety, as well as strategies for their reduction. The current analysis extends this model by confirming the significant role of gender, sensory factors, and medication use in shaping anxiety levels.

**Figure 7:**
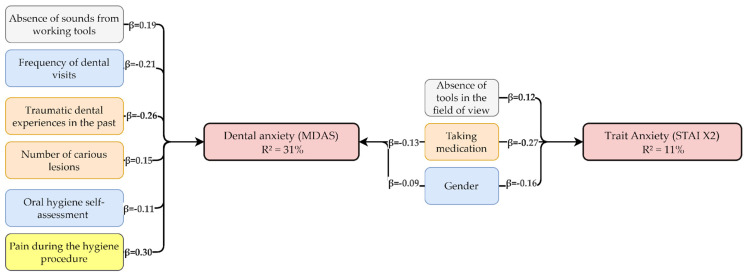
Multivariate regression model illustrating the influence of socio-demographic, clinical, and sensory factors on dental anxiety (MDAS) and trait anxiety (STAI X2), considering gender differences.

Additionally, a multivariate regression model (Figure 7) was used to estimate the influence of key variables such as gender, pain experienced during the procedure, stressful past dental experiences, medication use, and sensory factors on levels of dental anxiety (MDAS) and trait anxiety (STAI X2).

The combination of the conceptual model and the results of the multivariate regression analysis strengthens the understanding of mechanisms influencing the experience of fear and anxiety, which can support the design of effective strategies for their reduction.

In clinical settings, these insights can assist dental teams in tailoring care to individual needs, ultimately improving comfort and reducing anxiety during hygiene visits.

### 4.7. Limitations of the Conducted Study

When analyzing the obtained results, it is important to consider the limitations of the conducted study. These include the sample size and the uneven distribution of women (147) and men (100), which is not ideally balanced. A larger sample could provide more precise data and increase the statistical power of the results. Future studies should aim to recruit more gender-balanced samples and possibly expand the participant pool to enhance generalizability. Although the study used widely recognized tools for measuring anxiety, such as the MDAS, STAI X1, and STAI X2 scales, which are highly reliable, it should be noted that they are based on subjective responses from participants. Self-assessments may be prone to errors related to individual interpretation of questions, the influence of temporary mood, or a tendency to respond in ways that align with social expectations.

To strengthen the objectivity of future results, researchers could include physiological or clinician-rated measures alongside self-report questionnaires. Another limitation is that the study group came from a homogenous demographic background, which may make it difficult to generalize the results to populations with diverse cultural, social, or economic backgrounds. Future research should include more diverse populations to assess whether the observed patterns hold across different socio-cultural contexts. The lack of long-term follow-up is also a significant limitation. Conducting an analysis of the long-term effects of dental procedures on anxiety levels or other psychological reactions could provide a more comprehensive understanding of the impact of these procedures on patients. Longitudinal studies would allow for the assessment of whether anxiety responses persist, decrease, or increase over time following dental treatment.

Additionally, an important limitation of the study is the pronounced asymmetry observed between women and men regarding mental health problems and the use of medications. This disparity, with mental health issues being more prevalent in women and medication use higher in women, could influence the study results. While it was not feasible to modify the analysis at this stage, we acknowledge that this could be a confounding factor. Future research could address this by subsetting participants to control for these variables, allowing for a more accurate comparison between groups without these confounding factors. This would help corroborate the conclusions and minimize the impact of such imbalances.

### 4.8. Recommendations for Future Research

Recommendations for future research include involving a larger and more diverse sample to improve the generalizability of the results across different populations. It would also be beneficial to consider using more objective measurement tools, such as stress biomarkers (e.g., cortisol levels), to complement self-assessed anxiety measures. Long-term studies should also be conducted to examine the lasting effects of Guided Biofilm Therapy on dental anxiety. Additionally, it would be valuable to include other psychological factors, such as overall stress levels, in future research. Furthermore, integrating personalized treatment strategies into future studies could enhance the precision of dental anxiety management. Machine learning models and AI-driven analytics may help predict individual anxiety responses, allowing for tailored patient care approaches. The development of dynamic, real-time biofeedback systems could provide a more objective assessment of a patient’s stress levels, enabling the implementation of customized anxiety reduction protocols based on physiological and psychological markers.

These personalized approaches align with the broader trend of precision dentistry, ensuring that interventions are tailored to each patient’s unique needs rather than relying on generalized treatment protocols.

At the same time, future studies should aim to address the observed gender differences, particularly the asymmetry between women and men regarding mental health problems and medication use. These factors could significantly influence the results, and controlling for them in future research would provide more accurate comparisons between genders. Subgroup analyses excluding participants with mental health issues or medication use could help corroborate findings and reduce the impact of these confounding factors.

## 5. Conclusions

This study showed that during hygiene procedures utilizing GBT technology, women experienced higher levels of dental anxiety (MDAS) and trait anxiety (STAI X2) compared to men. These findings emphasize the need to consider gender differences when developing strategies to reduce anxiety during dental hygiene visits.

The multivariate regression models accounted for 11% of the variance in trait anxiety (STAI X2) and 31% of the variance in dental anxiety (MDAS), identifying gender as a relevant predictor in both models. Other factors, such as sensory stimuli and previous traumatic dental experiences, also played a role in shaping anxiety levels.

Practical implications include adopting tailored approaches to address gender-specific needs, such as providing relaxation techniques, allowing breaks during procedures, ensuring communication with staff after visits, and minimizing sensory discomfort (e.g., eliminating tool sounds). These measures could help reduce anxiety, particularly among women, improve patient comfort, and encourage more consistent participation in dental hygiene care.

A personalized approach to anxiety management may further enhance patient satisfaction and treatment adherence, aligning with the principles of patient-centered dental care. By implementing these adjustments, patients may feel more at ease during visits, which could reduce avoidance behaviors and lead to better oral and overall health outcomes.

## Figures and Tables

**Table 1:** Sociodemographic and clinical characteristics of study participants (n = 247).

Characteristic	Value	
	Women	Men
	Mean ± SD	
Age [years]	41.70 ± 17.06	45.49 ± 16.32
	n (%)	
Number of participants	147 (59.51)	100 (40.49)
Age [years]		
18–34	45 (30.60)	34 (34.00)
35–54	49 (33.30)	36 (36.00)
55–76	53 (36.10)	30 (30.00)
Education		
primary/middle school/secondary	36 (24.00)	34 (34.00)
Higher	111 (76.00)	66 (66.00)
First-time hygiene visit		
Yes	14 (9.52)	12 (12.00)
No	133 (90.47)	88 (88.00)
Orthodontic treatment in the past		
Yes	62 (42.18)	33 (33.00)
No	85 (57.82)	67 (67.00)
Dental visits in childhood		
Yes	137 (93.20)	86 (86.00)
No	10 (6.80)	14 (14.00)
Physical health problems		
Yes	48 (32.65)	20 (20.00)
No	99 (67.35)	80 (80.00)
Mental health problems		
Yes	16 (10.88)	2 (2.00)
No	131 (89.12)	98 (98.00)
Diagnosis of dentophobia		
Yes	0 (0.00%)	0 (0.00%)
No	147 (100.00%)	100 (100.00%)

Note: SD—Standard deviation.

**Table 2:** Levels of fear, anxiety and heart rate before and after the hygiene procedure.

Variable	Before Procedure (Mean ± SD)		Test Result	*p*-Value	After Procedure (Mean ± SD)		Test Result	*p*-Value
	Women	Men			Women	Men		
Dental anxiety (MDAS)	9.01 ± 3.86	7.83 ± 2.92	Z= −2.43	0.015	-	-	-	-
Trait anxiety (STAI X2)	45.74 ± 3.95	43.98 ± 4.16	Z = −3.13	0.002	-	-	-	-
State anxiety (STAI X1)	45.77 ± 4.17	45.81 ± 4.33	Z = 0.39	NS	44.20 ± 4.10	43.16 ± 4.10	Z = −1.96	NS
Dental fear (Gatchel’s scale)	3.86 ± 2.50	3.35 ± 2.12	Z = −1.32	NS	3.21 ± 2.31	2.77 ± 2.00	Z = −1.32	NS
Fear related to the hygiene procedure (author-developed question)	2.67 ± 2.19	2.27 ± 1.65	Z = −0.81	NS	2.12 ± 1.69	1.91 ± 1.28	Z = −0.06	NS
Heart rate [bpm] *	76.37 ± 8.01	74.39 ± 9.38	Z = −1.33	NS	74.82 ± 7.88	71.94 ± 7.77	Z = −2.55	0.0108

Note: SD—standard deviation, Z—Mann-Whitney U test standardized statistic, NS—not statistically significant. * Heart rate was also measured during the procedure, Mann-Whitney U test result: Z = −0.20, p: NS.

**Table 3:** Level of dental anxiety in men and women.

Gender	Mild Anxiety	Moderate Anxiety	High Anxiety	Severe Anxiety
Women *n* (%)	60 (40.82%)	37 (25.17%)	20 (13.61%)	30 (20.41%)
Men *n* (%)	55 (55.00%)	19 (19.00%)	15 (15.00%)	11 (11.00%)

**Table 4:** Trait anxiety levels (STAI X2) in women and men.

Gender	No Anxiety	Mild Anxiety	Moderate Anxiety	Severe Anxiety
Women *n* (%)	0 (0.00)	30 (20.41%)	112 (76.19%)	5 (3.40%)
Men *n* (%)	0 (0.00)	37 (37.00%)	61 (61.00%)	2 (2.00%)

**Table 5:** Factors that, in the patient’s opinion, could reduce negative emotions in women and men (The means are based on a 4-point scale: 0—definitely not, 1—probably not, 2—probably yes, 3—definitely yes).

Factors That, in the Patient’s Opinion, Could Reduce Negative Emotions	(Mean ± SD)		Test Result	*p*-Value
	Women	Men		
Absence of tools in the field of view	1.64 ± 0.91	1.21 ± 0.98	Z = −3.46	0.001
Absence of sounds from working tools	1.60 ± 0.90	1.28 ± 0.92	Z = −2.53	0.012
Absence of characteristic dental office smells	1.61 ± 0.92	1.29 ± 1.01	Z = −2.46	0.014
Possibility of using sedatives and painkillers	1.97 ± 0.88	1.69 ± 0.93	Z = −2.20	0.028
Possibility of taking breaks according to the patient’s needs	2.33 ± 0.78	2.04 ± 0.72	Z = −3.00	0.003
Possibility of contacting the clinic/dentist/hygienist	2.69 ± 0.57	2.48 ± 0.61	Z = −2.62	0.009

Note: SD—standard deviation, Z—Mann-Whitney U test standardized statistic.

**Table 6:** Selected clinical factors in women and men.

Variable	Women *n* (%)	Men *n* (%)	Test Result	*p*-Value
Physical health problems	48 (32.65)	20 (20.00)	Chi^2^ = 4.78	0.029
Mental health problems	16 (10.88)	2 (2.00)	Chi^2^ = 6.95	0.009
Taking medication	67 (45.58)	26 (26.00)	Chi^2^ = 9.72	0.002
Thyroid medications	25 (17.01)	2 (2.00)	Chi^2^ = 13.77	0.000
Medications for depression, insomnia, and other mental disorders (psychotropic drugs)	14 (9.52)	2 (2.00)	Chi^2^ = 5.56	0.018
“Other” medications	19 (12.93)	3 (3.00)	Chi^2^ = 7.23	0.007

Note: Chi^2^—Chi Pearson.

**Table 7:** The relationship between Dental Anxiety (MDAS) and Trait Anxiety (STAI X2) and taking medications.

Variable	Thyroid Medications		Medications for Depression, Insomnia, and Other Mental Disorders (Psychotropic Drugs)		“Other” Medications	
	Test Result	*p*-Value	Test Result	*p*-Value	Test Result	*p*-Value
Dental Anxiety (MDAS)	Chi^2^ = 1.16	NS	Chi^2^ = 26.13	<0.0001	Chi^2^ = 5.65	NS
Trait Anxiety (STAI X2)	Chi^2^ = 5.96	0.0507 (MS)	Chi^2^ = 0.57	NS	Chi^2^ = 11.59	0.003

Note: Chi^2^—Pearson’s Chi-square, NS—not statistically significant, MS—marginal significance.

**Table 8:** Multivariate Regression Analysis for Predicting STAI X2 Scores.

Variable	R^2^	β	F	*p*-Value
**Step 1**	0.07		19.12	<0.0001
Taking medication		−0.27		<0.0001
**Step 2**	0.10		13.26	<0.0001
Taking medication		−0.24		0.000
Gender		−0.16		0.009
**Step 3**	0.11		10.19	<0.0001
Taking medication (1-yes, 2-no)		−0.23		0.000
Gender (1-woman, 2-man)		−0.14		0.031
Absence of tools in the field of view		0.12		MS

Note: R^2^—coefficient of determination, β—standardized regression coefficient, MS—marginal significance.

**Table 9:** Multivariate Regression Analysis for Predicting MDAS Scores.

Variable	R^2^	β	F	*p*-Value
**Step 1**	0.10		28.64	<0.0001
Pain experienced during hygiene procedures		0.32		<0.0001
**Step 2**	0.17		25.69	<0.0001
Pain experienced during hygiene procedures		0.30		<0.0001
Traumatic dental experiences in the past		−0.26		<0.0001
**Step 3**	0.21		21.56	<0.0001
Pain experienced during hygiene procedures		0.30		<0.0001
Traumatic dental experiences in the past		−0.25		<0.0001
Absence of sounds from working tools		0.19		0.001
**Step 4**	0.25		20.46	<0.0001
Pain experienced during hygiene procedures		0.27		<0.0001
Traumatic dental experiences in the past		−0.25		<0.0001
Absence of sounds from working tools		0.22		0.000
Frequency of dental visits		−0.21		0.000
**Step 5**	0.28		18.30	<0.0001
Pain experienced during hygiene procedures		0.26		<0.0001
Traumatic dental experiences in the past		−0.26		<0.0001
Absence of sounds from working tools		0.22		0.000
Frequency of dental visits		−0.19		0.001
Number of carious lesions		0.15		0.007
**Step 6**	0.29		16.47	<0.0001
Pain experienced during hygiene procedures		0.27		<0.0001
Traumatic dental experiences in the past		−0.26		<0.0001
Absence of sounds from working tools		0.21		0.000
Frequency of dental visits		−0.19		0.001
Number of carious lesions		0.14		0.009
Taking medication		−0.13		0.019
**Step 7**	0.30		14.77	<0.0001
Pain experienced during hygiene procedures		0.24		<0.0001
Traumatic dental experiences in the past		−0.25		<0.0001
Absence of sounds from working tools		0.20		0.000
Frequency of dental visits		−0.17		0.0029
Number of carious lesions		0.13		0.0162
Taking medication		−0.12		0.0317
Oral hygiene self-assessment		−0.11		NS (0.0617)
**Step 8**	0.31		13.37	<0.0001
Pain experienced during hygiene procedures		0.24		<0.0001
Traumatic dental experiences in the past (1-yes, 2-no)		−0.25		<0.0001
Absence of sounds from working tools		0.19		0.0010
Frequency of dental visits (1–rare, 2–normal, 3–frequent		−0.17		0.0030
Number of carious lesions		0.13		0.0171
Taking medication (1-yes, 2-no)		−0.10		NS (0.0744)
Oral hygiene self-assessment (scale 0–4, 0–very poor, 4–very good)		−0.12		0.0448
Gender (1-woman, 2-man)		−0.09		NS (0.0960)

Note: R^2^—coefficient of determination, β—standardized regression coefficient, NS—not statistically significant.

## Data Availability

Data are available on request due to privacy/ethical restrictions.
